# How Does the Intricate Mouthpart Apparatus Coordinate for Feeding in the Hemimetabolous Insect Pest *Erthesina fullo*?

**DOI:** 10.3390/insects11080503

**Published:** 2020-08-04

**Authors:** Yan Wang, Wu Dai

**Affiliations:** Key Laboratory of Plant Protection Resources and Pest Management of the Ministry of Education, College of Plant Protection, Northwest A&F University, Yangling 712100, China; wangyan105422@163.com

**Keywords:** *Erthesina fullo*, mouthparts, sensillum, ultramorphology, feeding performance

## Abstract

**Simple Summary:**

To better understand the feeding mechanism of *Erthesina fullo*, the fine structure of the mouthparts is examined with scanning electron microscopy, and feeding performance are observed directly under laboratory conditions for the first time. The adult feeding process involves several steps, including exploring and puncturing of the host plant epidermis, a probing phase, an engorgement phase, and removal of the mouthparts from the host tissue. Proceeding from labium towards the mandibular stylets, the movement pattern becomes increasingly stereotypical, including the sensilla on the tip of the labium probing, the labium making an elbow-like bend between the first and second segment, the base of the stylet fascicle housing in the groove of the labrum, the mandibular stylets penetrating the site and maxillary stylets feeding. The morphology of mouthparts is similar to those of other Heteroptera. The four-segmented labium has eleven types of sensilla. The mandibular stylet tips have two nodules preapically on the convex external surface. The structure and function of the mouthparts are adapted for the phytophagous feeding habit in this species. This study increases the available detailed morphological and behavioral data for Hemiptera and will potentially contribute to improving our understanding of this pest’s feeding behavior and sensory mechanisms.

**Abstract:**

The yellow marmorated stink bug, *Erthesina fullo* (Thunberg, 1783), is a major pest of certain tree fruits in Northeast Asia. To better understand the feeding mechanism of *E. fullo*, the fine structure of the mouthparts, including the distribution and abundance of sensilla, are examined with scanning electron microscopy (SEM), and their functions are observed directly under laboratory conditions. The feeding performance is described in detail and illustrated for the first time. The adult feeding process involves several steps, including exploring and puncturing of the host plant epidermis, a probing phase, an engorgement phase, and removal of the mouthparts from the host tissue. Proceeding from labium towards the mandibular stylets, the movement pattern becomes increasingly stereotypical, including the sensilla on the tip of the labium probing, the labium making an elbow-like bend between the first and second segment, the base of the stylet fascicle housing in the groove of the labrum, the mandibular stylets penetrating the site and maxillary stylets feeding. In terms of morphology, the mouthparts are similar to those of other Heteroptera, consisting of a triangular pyramidal labrum, a tube-like and segmented labium with a deep groove on the anterior side, and a stylet fascicle consisting of two mandibular and two maxillary stylets. The four-segmented labium has five types of sensilla basiconica, three types of sensilla trichodea, two types of sensilla campaniformia and 1 type of sensilla coeloconica. Among them, sensilla trichodea one and sensilla basiconica one are most abundant. The tripartite apex of the labium is covered with abundant sensilla trichodea three and a few sensilla basiconica 5. The mandibular stylet tips have two nodules preapically on the dorsal margin of the convex external surface, which may help in penetrating plant tissue and anchoring the mouthparts. The externally smooth maxillary stylets interlock to form a larger food canal and a smaller salivary canal. The structure and function of the mouthparts are adapted for the phytophagous feeding habit in this species. Similarities and differences between the mouthparts of *E. fullo* and those of other Heteroptera are discussed.

## 1. Introduction

Hemiptera is the largest and most diverse non-holometabolous insect order, containing over 75,000 species. They are characterized by specialized piercing-sucking mouthparts, in which the modified mandibles and maxillae form two pairs of stylets sheathed within a modified labium [1,2]. These mouthparts facilitate feeding on fluids of various animal and plant hosts and have sensory organs used in both host location and feeding. The Hemiptera have been classified into four major taxa (suborders: Auchenorrhyncha, Sternorrhyncha, Coleorrhyncha and Heteroptera). Abundant data are available on some aspects of hemipteran mouthpart morphology based on light and scanning electron microscopy, for a few species of Auchenorrhyncha, e.g., Fulgoroidea [3,4], Cicadellidae [5,6,7,8,9], Aphidoidea [10,11,12,13], Coccoidea [14] and Aleyrodidae [15,16] of Sternorrhyncha and the Heteroptera [17,18,19,20]. These provide insights into feeding mechanisms and contribute to assessment of phylogenetic relationships [7,8,11,13,19,21,22,23,24,25]. However, so far, mouthpart morphology of some major groups remains little studied.

As a biologically successful group of organisms, the heteropterans (true bugs) are prolific and diverse and have acquired a variety of feeding habits. Some heteropterans suck surface fluids (e.g., nectar), some pierce tissues to suck sap or blood, and others obtain nourishment from dried seeds. Numerous modifications of mouthpart structures reflect the diversity of food sources and feeding habits of this group. Cobben [17] studied the heteropteran feeding stylets of 57 families and 145 species, but provided little information on the labium and the types and distributions of sensilla present on this stucture. In the carnivorous heteropterans, different feeding mechanisms are reflected in differences in the labial tip sensilla [26] and the movement and penetration of the stylets during feeding [27].

The strategies used by various phytophagous Heteroptera to feed on a variety of plant structures may include stylet-sheath feeding, lacerate and flush feeding, macerate and flush feeding, and osmotic pump feeding [17,28]. In general, feeding damage from heteropterans can be classified into five categories: Localized wilting and necrosis, abscission of fruiting forms, morphological deformation of fruits and seeds, modified vegetative growth, and tissue malformation [29,30,31]. All plant-sucking heteropterans are potential vectors of plant disease, and the lesions left behind at the feeding site can facilitate secondary infections by plant pathogens [32].

Pentatomidae (stink bugs) is one of the largest families within the Heteroptera. Stink bugs feed by inserting their stylets into the food source to suck up nutrients and may transmit plant pathogens, resulting in plant wilt and, in many cases, abortion of fruits and seeds. Compared with more specialized Hemiptera, Pentatomidae use diverse feeding strategies that allow them to feed from a wide range of plant structures including vegetative structures, such as stems and leaves, and reproductive plant structures such as seeds, nuts, pods and fruits [33]. Stink bug feeding can damage crops in different ways dependent upon the plant structure(s) attacked, e.g., vegetative or reproductive. Previous studies of mouthparts in Pentatomidae have mostly focused upon differences in certain aspects of the mouthparts among stinkbug species, such as the types and distribution of labial sensilla [34,35,36] and the internal structure of mandibular and maxillary stylets [37]. Information on the distribution of sensilla on the mouthparts and the relationships between mouthpart structure and function in feeding, useful in the classification of stink bugs, is not yet available.

The yellow marmorated stink bug *Erthesina fullo* (Thunberg, 1783) is one of the most widely distributed phytophagous pests in East Asia. It causes severe loss to many horticultural crops, such as apples, cherries and pears [38,39,40], and disturbs humans by invading houses in large numbers when overwintering. Both nymphs and adults of *E. fullo* primarily suck the sap from the trunk, leaves, immature stems and fruits of plants. Previous research on this species has mainly been limited to basic biology, behavior and integrated control [39,41,42]. Although, feeding damage from *E. fullo* has been characterized in agronomic crops, tree fruits and vegetables, little is known about the fine structure of the mouthparts and the feeding mechanism of *E. fullo,* and in particular, how the sensilla are distributed on the mouthparts and function in locating host plants.

Here, we used scanning electron microscopy to investigate the mouthpart morphology and distribution of sensilla of *E. fullo*. We also observed feeding behavior. The outcome of this study increases the available detailed morphological and behavioral data for Hemiptera and will potentially contribute to improving our understanding of this pest’s feeding behavior and sensory mechanisms. This study provides more data for future comparative morphological studies in Pentatomidae.

## 2. Material

### 2.1. Insect Collecting

Adults of *E. fullo* used for SEM in this study were obtained from the campus of Northwest A&F University in Yangling, Shaanxi Province, China (34°16′ N, 108°07′ E, elev. 563 m) in August 2016, and were preserved in 70% ethanol and stored at 4 °C. For observing feeding behavior, additional adults of *E. fullo* were collected at the same locality in September 2019.

### 2.2. Samples for SEM

Ten females and twelve male specimens of *E. fullo* were fixed in 70% ethanol. The labium and the stylet, dissected using fine dissecting needles under 40× magnification (Nikon SMZ 1500, stereomicroscope, Tokyo, Japan), were prepared as study samples. The samples were cleaned in an ultrasonic bath (250 W) (KQ118, Kunshan, China) for 10 to 15 s in 70% ethanol three times, then dehydrated in serial baths of 80%, 90% and 100% ethanol each for 15 min. Samples then underwent dehydration in a mixture of 100% ethanol and 100% tert-Butanol at the ratios 3:1, 1:1, and 1:3 (by volume) for 15 min at each concentration followed by a final replacement treatment in 100% tert-Butanol for 30 min. Specimens were then freeze-dried with liquid CO_2_, mounted on aluminum stubs with double-sided copper sticky tape and sputtered with gold/palladium (40/60) in a LADD SC-502 (Vermont, USA) high resolution sputter coater. The samples were subsequently examined with a Hitachi S-3400N SEM (Hitachi, Tokyo, Japan) operated at 15 kV [8] or Nova Nano SEM-450 (FEI, Hillsboro, OR, USA) at 5–10 kV.

### 2.3. Feeding Behavior on Different Types of Substrates

To observe the feeding behavior of the insects on fresh fruit and twigs, some fruit of orange, pear and grape as well as twigs of pear and grape were offered to twenty male and female individuals of *E. fullo* in an optical quality colorless glass enclosure 100 mm in diameter and 135 mm tall. The insects were observed intermittently throughout the feeding period for one week. Sequential images of adult feeding performance were taken using a mobile phone (vivo Y18L) with an 8-megapixel camera when conditions were suitable. The images were saved directly to a computer for later analysis.

### 2.4. Image Processing and Terminology

Photographs and SEMs of mouthparts were observed and measured after being imported into Adobe Photoshop CC 2019 (Adobe Systems, San Jose, CA, USA). Measurements are given as means ± standard error of the mean. Schematic diagrams were drawn with Microsoft Office Word 2007 and processed with Photoshop CC 2019 (Adobe Systems, San Jose, CA, USA). For classification of sensilla, the systems of Altner and Prillinger [43] were used in addition to the more specialized nomenclature from other studies [44].

### 2.5. Data Analysis

The lengths of the mouthparts were compared between sexes using a Student t-test. Statistical analyses were executed using SPSS 19.0 (SPSS, Chicago, IL, USA).

## 3. Results

### 3.1. General Morphology and Structure of Mouthparts

**Figure 1:**
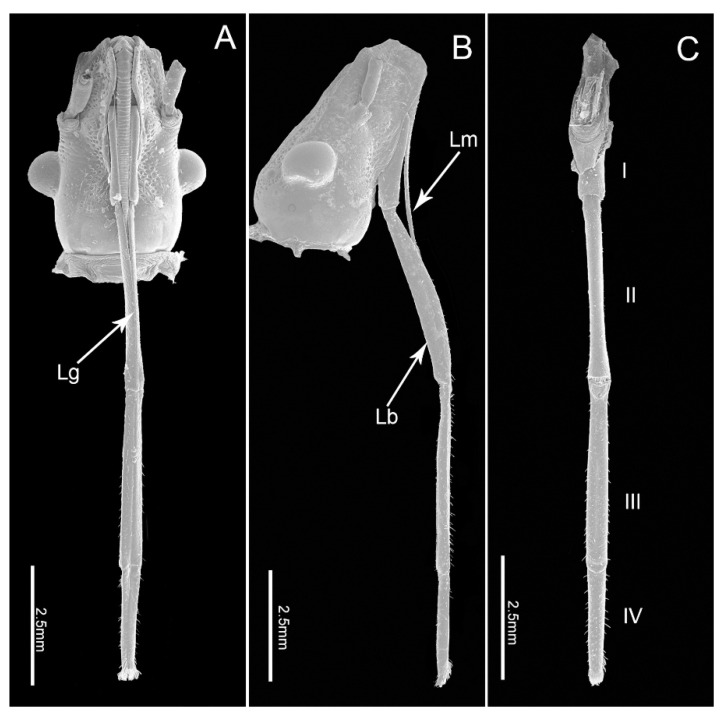
Scanning electron micrographs of the head of *E fullo*. (**A**) Ventral view; (**B**) Lateral view; (**C**) Dorsal view showing four-segmented labium (I–IV); Lg, labial groove; Lm, labrum; Lb, labium.

The mouthparts of *E. fullo* are similar to those of other heteropterans, arising from the anteroventral part of the head capsule and composed of a long labrum, a tube-like labium and a stylet bundle comprising two maxillary stylets (Mx) and two mandibular stylets (Md). The four-segmented labium has a long internal labial groove (Lg) that surrounds the stylet fascile (Sf) and is covered with different types of sensilla symmetrically distributed on the surface of both sides of the groove or on the distal surface (Figure 1A–C). The two inner maxillary stylets interlock to form the food and salivary canals; they are partially surrounded by two serrate-edged mandibular stylets. The stylet fascicle is housed inside the labial groove and proximally covered by the small cone-shaped labrum. No obvious differences were noted between the mouthpart structures of females and males except for the length of the labium (t(9) = 9.473, *p* = 0.000) (Table 1).

#### 3.1.1. Labrum

**Figure 2:**
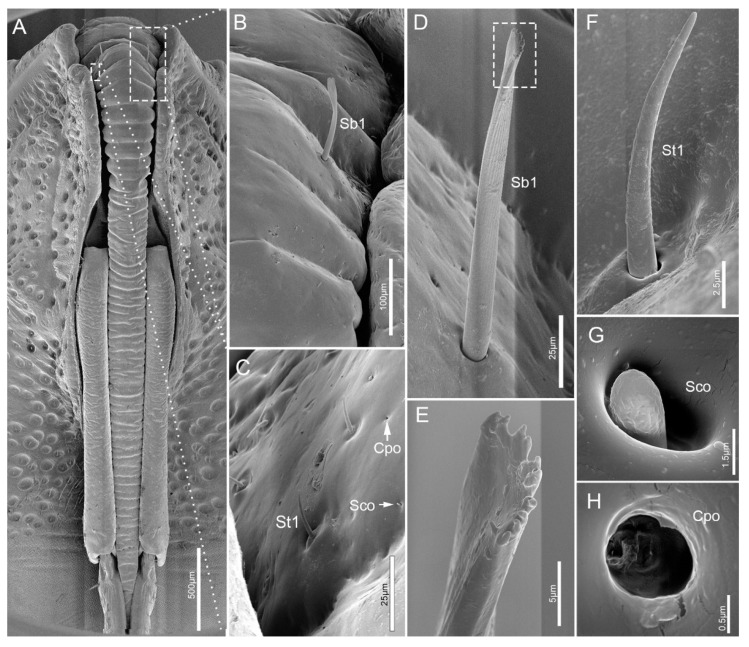
SEM of labrum of *E. fullo*. (**A**) Ventral view; (**B**) Enlarged view of box in (**A**), showing sensillum basiconicum 1 (Sb1); (**C**) Enlarged view of box in (**A**), showing sensilla trichodea 1 (St1), sensilla coeloconica (Sco) and cuticular pores (Cpo); (**D**) Sensillum basiconicum 1 (Sb1); (**E**) Enlarged view of box in (**D**); (**F**) Sensillum trichodeum 1 (St1); (**G**) Sensillum coeloconicum (Sco); (**H**) Enlarged view of cuticular pore (Cpo).

The cone-shaped labrum (Lm) is attached to the anterior margin of the anteclypeus and protrudes forward beyond 2/5 length of the second segment (Figure 1A and Figure 2A). It is closely adpressed over the first labial segment and partly embedded in the labial groove. The surface of the labrum is plicated and densely covered with regular transverse wrinkles (Figure 2A). The ventral side of the labrum bears a pair of sensilla basiconica 1 (Sb1). Sensilla trichodea 1 (St1), sensilla coeloconica (Sco) and cuticular pores (Cpo) are arranged irregularly on the ventral region of the labrum (Figure 2A–H).

#### 3.1.2. Labium

The labium, suspended from the front of the head, is tubular in shape and subdivided into four-segments externally (Figure 1A–C). The anterior surface of the labium is bisected by a deep longitudinal groove, which encases the mandibular and maxillary stylets. All segments of the labium are covered with different types of sensilla mainly positioned symmetrically on each side of the labial groove (Lg) and distally, with fewer sensilla on the posterior and lateral surfaces.

The four segments vary in size (Table 1) and morphology (Figure 1A–C). Overall the labium is broad and of uniform width through most of its length with the distal segment widening near the tip.

**Figure 3:**
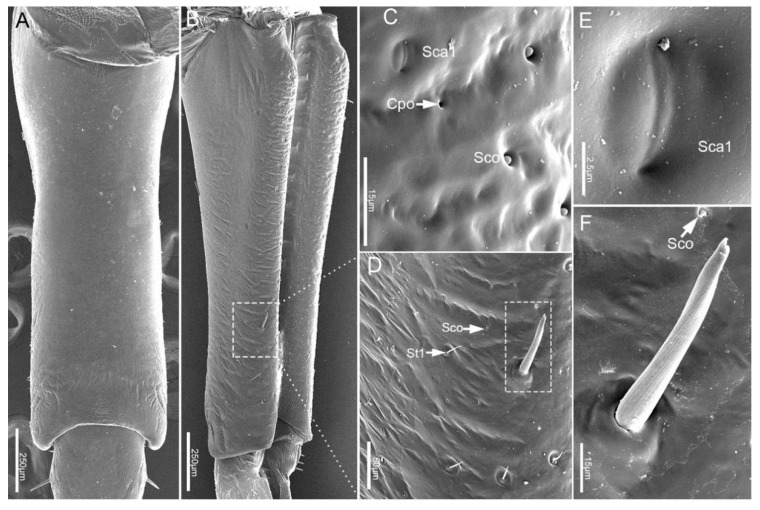
SEM of first labial segment of *E. fullo*. (**A**) Ventral view; (**B**) Lateral view; (**C**) Enlarged view of surface of the first segment, showing sensilla campaniformia 1 (Sca1), sensilla coeloconica (Sco) and cuticular pores (Cpo); (**D**) Enlarged view of outlined box in (**B**), showing sensilla trichodea 1 (St1), sensilla coeloconica (Sco) and sensilla basiconica 1 (Sb1); (**E**) Sensillum campaniformium 1 (Sca1); (**F**) Enlargement of outlined box in (**D**), showing sensillum coeloconicum (Sco) and sensillum basiconicum 1 (Sb1).

The proximal labial segment (Lb1), the shortest and widest of the four segments, is broad at the base, gradually narrows at the middle, and then slightly widens to the apex in posterior view (Figure 2A and Figure 3A,B). The distal part of the dorsum is contracted inward, is crescent-shaped and no sensilla are observed on this surface (Figure 3A). Four types of sensilla (sensilla basiconica 1, sensilla trichodea 1, sensilla coeloconica, sensilla campaniformia 1) and cuticular pores (Cpo) are arranged on the ventral surface (Figure 3B–F).

**Figure 4:**
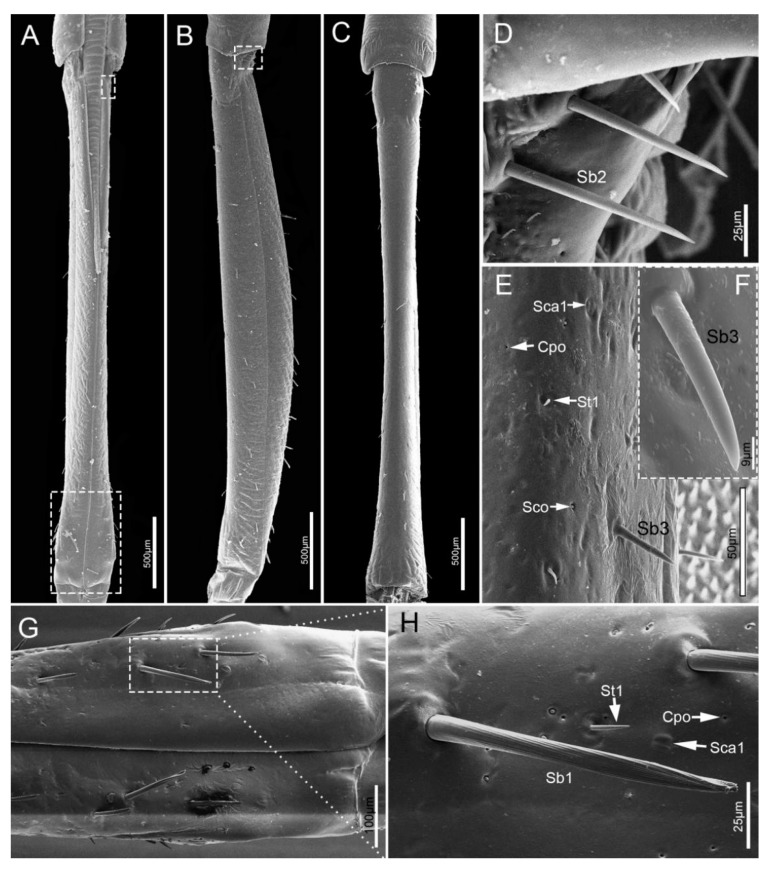
SEM of second labial segment of *E. fullo*. (**A**) Ventral view; (**B**) Lateral view; (**C**) Dorsal view; (**D**) Enlarged view of outlined box of (**B**) showing sensilla basiconica 2 (Sb2); (**E**) Enlarged view of outlined box of (**A**) showing sensilla campaniformia 1 (Sca1), cuticular pores (Cpo), sensilla trichodea 1 (St1), sensilla coeloconica (Sco) and sensilla basiconica 3 (Sb3); (**F**) Sensillum basiconicum 3 (Sb3); (**G**) Enlarged view of surface of the second segment of outlined box of (**A**); (**H**) Enlarged view of outlined box of (**G**), showing sensilla basiconica 1 (Sb1), sensilla trichodea 1 (St1), cuticular pore (Cpo) and sensilla campaniformia 1 (Sca1).

The second segment (Lb2) is longer than the first segment (Table 1). Viewed from the ventral and dorsal sides, the base and ends are wider, while the middle part is narrower (Figure 4A,C). However, from the lateral view, the middle part is expanded, and both ends are narrowed (Figure 4B). Six types of sensilla were found on this segment, including three types of sensilla basiconica (Sb1, Sb2, Sb3), one type of sensilla campaniformia (Sca1), and one type of sensilla trichodea (St1) and sensilla coeloconica (Sco) (Figure 4D–H). Also, there are some cuticular pores (Cpo) arranged on the surface of second segment (Figure 4E).

**Figure 5:**
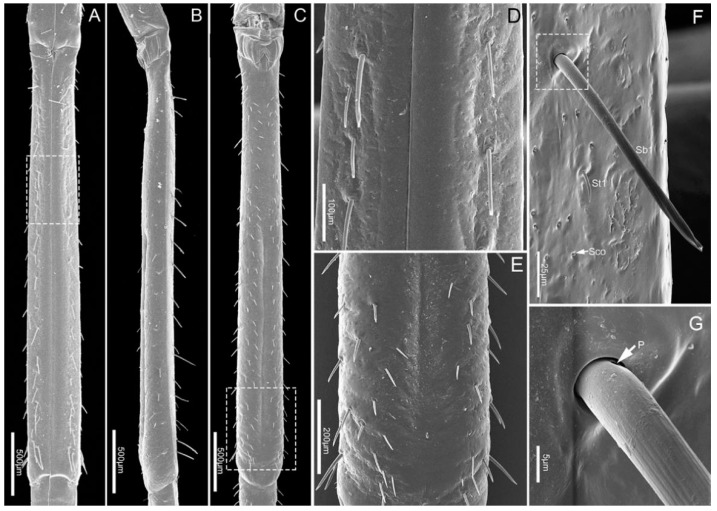
SEM of the third labial segment of *E. fullo*. (**A**) Ventral view; (**B**) Lateral view; (**C**) Dorsal view; (**D**) Enlarged view of outlined box of (**A**); (**E**) Enlarged view of outlined box of (**C**); (**F**) Enlarged view of surface of the third segment, showing sensilla basiconica 1 (Sb1), sensilla trichodea 1 (St1), and sensilla coeloconica (Sco); (**G**) Enlarged view of outlined box of (**F**), showing base pore (p).

The third segment (Lb3) is a little longer than the second, and of uniform width on both sides (Table 1, Figure 5A–C). Generally, there is a groove on the dorsal surface of the last 3/5 (Figure 5C). A wrinkled area is present on the dorsal surface at the internode between the second and third labial segment. Three types of sensilla are distributed on this part, including sensilla basiconica 1 (Sb1), sensilla trichodea (St1) and sensilla coeloconica (Sco) (Figure 5D–G).

**Figure 6:**
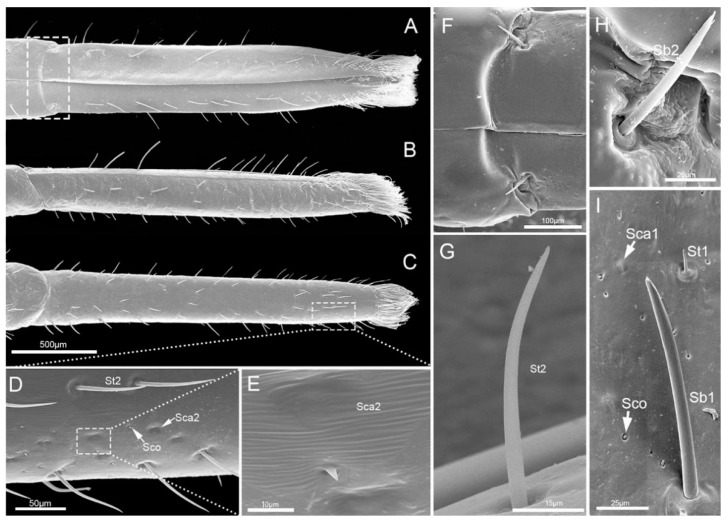
SEM of the fourth labial segment of *E. fullo*. (**A**) Ventral view; (**B**) Lateral view; (**C**) Dorsal view; (**D**) Enlarged view of outlined box of (**C**), showing sensilla trichodea 2 (St2), sensilla coeloconica (Sco) and sensilla campaniformia 2 (Sca2); (**E**) Enlarged view of outlined box of (**D**), showing sensilla campaniformia 2 (Sca2); (**F**) Enlarged view of outlined box of (A), showing sensilla basiconica 2 (Sb2); (**G**) Sensillum trichodeum 2 (St2); (**H**) Sensillum basiconicum 2 (Sb2); (**I**) Enlarged view of surface of the fourth segment, showing sensilla campaniformia 1 (Sca1), sensilla trichodea 1 (St1), sensilla coeloconica (Sco) and sensillum basiconicum 1 (Sb1).

**Figure 7:**
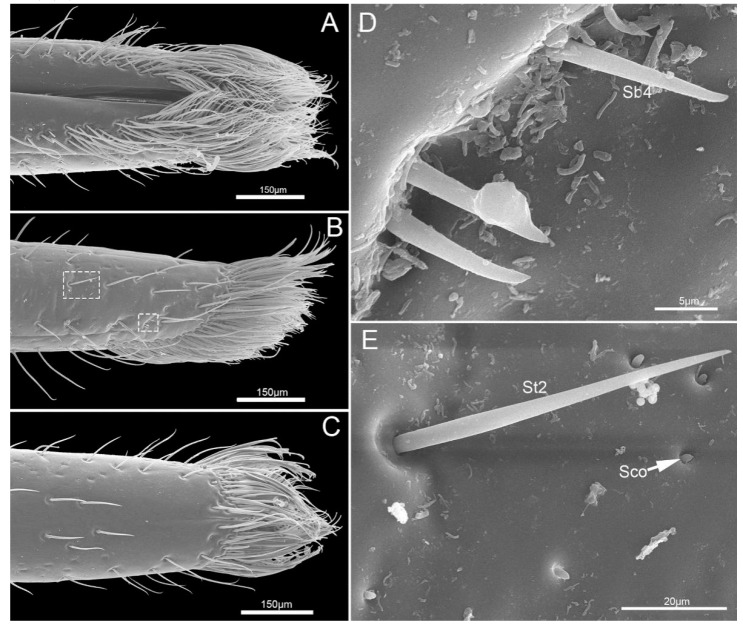
Proximal position the fourth labial segment of *E. fullo*. (**A**) Ventral view; (**B**) Lateral view; (**C**) Dorsal view; (**D**) Sensilla basiconica 4 (Sb4); (**E**) Enlarged view of outlined box of (**B**), showing sensillum trichodeum 2 (St2) and sensilla coeloconica (Sco).

**Figure 8:**
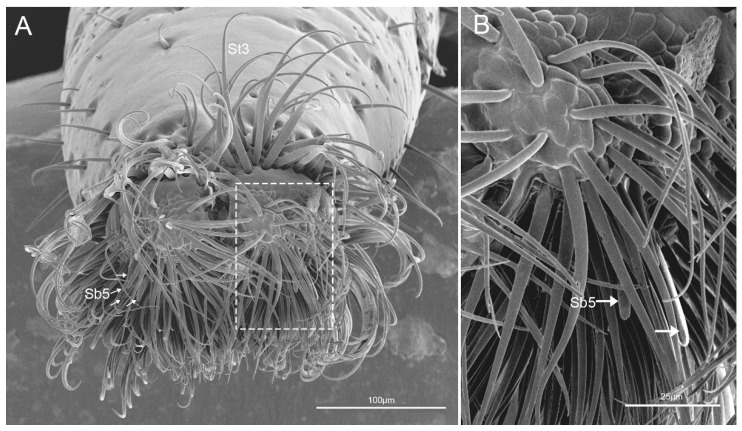
Tip of labium of *E.fullo*. (**A**) Vertical view of labial tip showing sensilla basiconica 5 (Sb5) and sensilla trichodea 3 (St3); (**B**) Enlarged view of outlined box of (**A**), showing sensilla basiconica 5 (Sb5).

The fourth segment (Lb4) is nearly conical, of uniform width from base to apical 1/4 then narrowing to the apex (Figure 6A–C). There are abundant sensilla distributed on this segment, including three types of sensilla trichodea (St1, St2, St3), three types of sensilla basiconica (Sb1, Sb2, Sb3), two types of sensilla campaniformia (Sca1, Sca2) and sensilla coeloconica (Sco) (Figure 6D–I and Figure 7A–E). *E. fullo* has very long and numerous sensilla trichodea 3 (St3) covering the end of the labium giving it a brush-like appearance (Figure 7A–C). Several sensilla basiconica 5 (Sb5) are visible among these sensilla trichodea 3 (St3) (Figure 8A,B).

#### 3.1.3. Labial Sensilla Types and Their Arrangement

Based on their external morphology and distribution, eleven types (subtypes based on the length and shapes are distinguished) of distinct sensilla were observed on the surfaces of the labial segments. They were classified as: sensilla trichodea (St), sensilla campaniformia (Sca), sensilla coeloconica (Sco) and sensilla basiconica (Sb).

Sensilla trichodea (St) are hair-like sensilla. Their walls are smooth without any pores or grooves on the surface. Three subtypes of sensilla trichodea were distinguished. Sensilla trichodea 1 (St 1) are short (Table 2), aporous, smooth, with a slightly rounded tip and flexible sockets (Figure 2F). These sensilla are numerous and uniformly distributed on the labrum (Lm) and labium (Lb1–4) (Figure 2C and Figure 3D). Sensilla trichodea 2 (St 2) are longer than sensilla trichodea 1 (Table 2), straight, with a smooth surface, a rounded tip and flexible sockets (Figure 6D,G). These sensilla are uniformly distributed on the fourth labial segment (Lb4). Sensilla trichodea 3 (St 3) are the longest sensilla (Table 2). These sensilla are curved at the tip and embedded in inflexible sockets. These sensilla are very numerous and located on the tip of the labium (Figure 8A,B).

Five subtypes of sensilla basiconica were distinguished. Sensilla basiconica 1 (Sb 1) are hair-like sensilla identical sensilla trichodea except for their smooth walls and blunt-tip. In the studied species, these sensilla are long (Table 2) ribbed and straight, slightly branched at the tip and arise from a cuticle with a flexible socket (Figure 2D,E). These sensilla are distributed on the labrum (Lm) and labium (Lb 2–4) (Figure 2B, Figure 3B,D,F, Figure 4G,H, Figure 5D–F and Figure 6I). Sensilla basiconica 2 (Sb 2) are cones with a smooth surface that arise from flexible sockets (Figure 4D and Figure 6H). Three pairs of sensilla basiconica 2 are arranged at the junction of the first and second segment, two are present on each side of the junction of the third and fourth segments (Figure 4D and Figure 6F). Sensilla basiconica 3 (Sb3) are short with a smooth surface, have a sharp tip and sit in a pit (Figure 4E,F). These sensilla are sparsely distributed on the ventral surface of the second segment. Sensilla basiconica 4 (Sb4) are peg-like with a smooth surface and have a rounded tip (Figure 7D). They are sparsely distributed on the lateral surface of the last segment (Figure 7B). Sensilla basiconica 5 (Sb 5) are present at the center of each distal lobe (Figure 7A). This type of sensillum is long, straight and with a smooth surface and a rounded tip, probably with a terminal pore (Figure 8B). Several of these sensilla basiconica are visible among the sensilla trichodea of the distal brush (Figure 7A,B).

Sensilla campaniformia (Sca) are flat, oval-shaped discs. Two subtypes of sensilla campaniformia are distinguished. Sensilla campaniformia 1 (Sca 1) are large (Table 2), numerous and present on the labrum (Lm) and labium (Lb 1–4) (Figure 2C, Figure 3E, Figure 4E,H and Figure 6I). Sensilla campaniformia 2 (Sca 2) are smaller than sensilla campaniformia 1 (Sca 1), fewer in number and located on the antero-lateral surface near the apical 1/3 (Figure 6D,E).

Sensilla coeloconica (Sco) consist of a small oval protuberance or cone inserted in a cuticular depression (Figure 2G). These are located on the labrum (Lm) and labium (Lb1–4) (Figure 2C, Figure 3C,D, Figure 4E, Figure 5F, Figure 6D,I and Figure 7E). These are without pores and have an inflexible socket.

### 3.2. Stylet Fascicle

**Figure 9:**
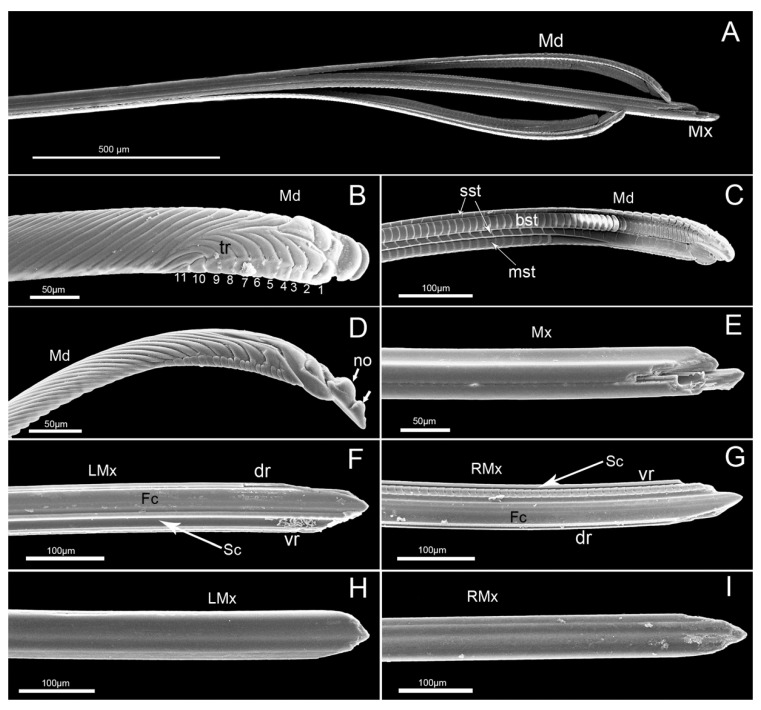
SEM of stylet fascicle of *E. fullo*. (**A**) Stylet fascicle showing mandibular (Md) and maxillary stylets (Mx); (**B**)External view of mandibular stylet (Md) showing eleven short transverse ridges (tr); (**C**) Interior view showing small squamous textures (sst), bigger squamous textures (bst) and middle squamous textures (mst); (**D**) Lateral view showing two nodules (no); (**E**) Apices of interlocked maxillary stylets; (**F**) Apex of left maxillary stylet (LMx) showing food canal (Fc) and salivary canal (Sc); (**G**) Apex of right maxillary stylet (RMx) showing food canal (Fc) and salivary canal (Sc); (**H**) External view of left maxillary stylet (LMx); (**I**) External view of right maxillary stylet (RMx); dr, dorsal side; vr, ventral side.

The stylet fascicle is long, slender, and composed of two separated mandibular stylets and two interlocked maxillary stylets (Figure 9A), ensheathed by the labium at rest and extending from the opening of the labial tip during feeding.

The mandibular stylets, located on each side of the maxillary stylets, are crescent-shaped in cross-section, convex externally and slightly concave internally to form a groove enclosing the maxillary stylets. On the lateral surface of each mandibular stylet, a series of approximately parallel, curved serrate ridges or teeth (a regular series of longer transverse ridges and eleven shorter transverse ridges) extend over the most distal part (Figure 9B). The most obvious features observed on the mandibular stylets of this species are two nodules present on the dorsal margin of the convex external surface near the apex (Figure 9B,D). There are four rows of squamous structures regularly distributed on the inner surface of the mandibular stylet (Figure 9C). The first and third rows consist of small squamous textures (sst), the second has bigger squamous textures (bst) and the fourth has medium-sized squamous textures (mst) with different cuticular spines.

**Figure 10:**
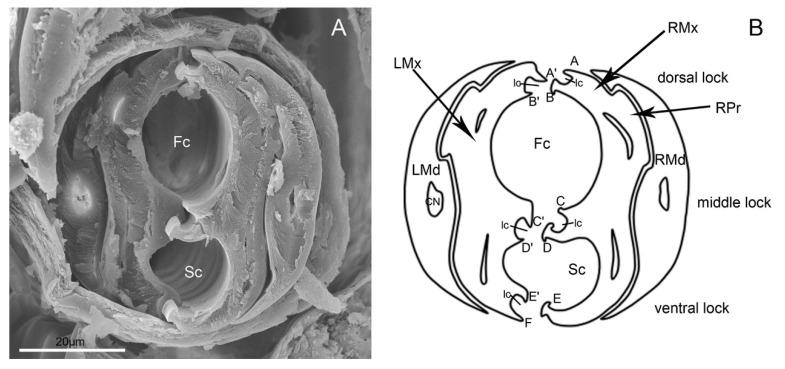
Cross-section of stylet fascicle of *E. fullo*. (**A**) Cross-section of stylet fascicle through middle of second and third segment showing food canal (Fc) and salivary canal (Sc); (**B**) Diagram of cross-section of stylet fascicle. LMd, left mandibular stylet; RMd, right mandibular stylet; LMx, left maxillary stylet; RMx, right maxillary stylet; Fc, food canal; Sc, salivary canal; Ic, interlocking canal; CN, dendritic canal; RPr, Right process of the maxilla; A, Straight; A’, Hooked; B, Hooked; B’, Straight; C, Straight; C’, Hooked; D, T-shaped; D’, Hooked; E, Hooked; E’, Hooked; F, Straight.

The maxillary stylets (Mx) are interlocked by hook-like hinges and are not symmetrical (Figure 9E). The hook-like hinges include three joints from the cross-section, one of which is located at the center of the maxillary stylets and two of which are positioned at the lateral sides (Figure 10A,B). The external and internal surface of a maxillary stylet is smooth and the tip is sharp (Figure 9F–I). A row of nodes is present on the joint surface of the left stylet, which opposes the series of indentations on the right stylet (Figure 9G). A food canal (Fc) and salivary canal (Sc) are formed by the interlocked maxillary stylets, and the width of food canals is evenly distributed across the two stylets, while most of the salivary canal is housed in the right stylet (Figure 9F,G). The diameter of the central food canal is much greater than that of the salivary canal (Figure 10A,B). The cross-section of the stylet fascicle shows that each mandibular stylet has a dendritic canal, which is a large duct that runs the length of the stylet and is located centrally in the thickest portion of each structure (Figure 10A,B).

### 3.3. The Process of Feeding by E. fullo

The adult feeding process involves several steps, including the exploring and puncturing of the plant epidermis, a probing phase, an engorgement phase, and removal of the mouthparts from the plant tissue. These processes vary slightly in mouthpart position and duration.

**Figure 11:**
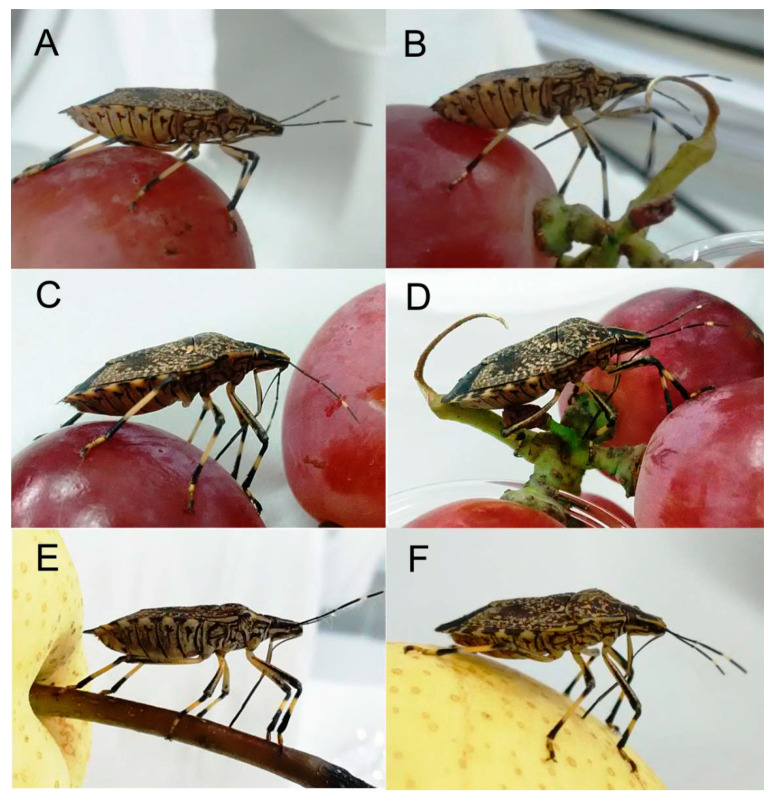
Feeding on fruits and young stalks in adult *E. fullo* showing positions of the mouthparts. (**A**) At rest or not feeding; (**B**) Exploring suitable feeding location; (**C**) Feeding on grape; (**D**) Feeding on green stalk of grape; (**E**) Feeding on stalk of pear; (**F**) Feeding on pear.

When the insect is at rest or not feeding, the rostrum is in contact with the ventral surface of the body from the front coxal base to the anterior part of the abdomen (Figure 11A). The proximal end of labial segment 2 articulates with the bucculae.

Insects feeding on the internal fluids of other organisms must first penetrate the plant tissues. After landing, an adult of *E. fullo* walks on its plant and explores for a suitable feeding location by probing. It then stops and remains still while the antennae swing up and down several times. After a few seconds, gripping the plant with its legs, the bug tilts the anterior part of its body upward at an angle to the surface, and the rostrum is then extended forward and used as a sense organ in conjunction with the eyes and antennae to examine the plant material for a suitable feeding spots. The labium first moves forward by swinging from its horizontal position of repose until it is perpendicular to the plant surface. The rostrum tip then taps the surface or slides over it. When the labium is moved forward from its resting position, the stylet tip reaches the tip of the labium and may even extend a short distance beyond the top (Figure 11B).

**Figure 12:**
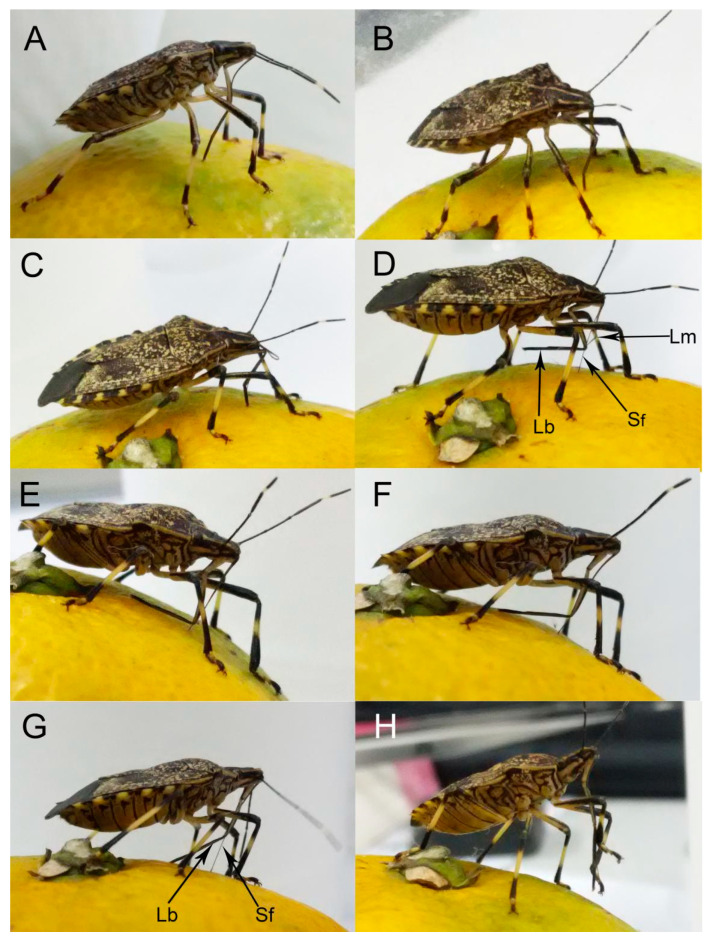
Feeding stages on orange in adult *E. fullo* showing positions of the mouthparts. (**A**) Location of suitable feeding position by the labium; (**B**), (**C**) Puncture of orange by stylet fascicle showing elbow-like fold of proximal and second rostral segments and stylet penetration; (**D**) The bug lifts up the third and fourth segments of labium parallel to host surface and then feeds; (**E**) The bug gradual straightens the first and second labial segments; (**F**), (**G**) Termination of feeding showing retraction of stylets; (**H**) Use of forelegs to return stylet fascicle to labial groove; Lm, labrum; Lb, labium; Sf, stylet fascicle.

Upon contact with a potential feeding site, the bug may probe with sensilla on the tip of the labium and penetrate the site with the stylets to test if this site is suitable for feeding. After selecting an appropriate feeding site, the insect then presses the tip of the labium onto the plant surface and inserts the feeding stylets. Then, the labium makes an elbow-like bend between the first and second segment, while the base of the stylet fascicle is held in the groove of the labrum (Figure 12A). The labium continues retracting to its maximum extent, at which the angle between the first and second segments is nearly 90°, allowing the head to be lowered as the stylet bundle penetrates the food tissue (Figure 12B,C), with the maxillary stylets lagging slightly behind the mandibular stylets. Stylet probing continues until a suitable tissue is found. It may take anywhere from five minutes to three hours from the beginning of probing until a feeding site is reached. The bug secretes viscous saliva as the stylets progress through the tissue.

After a feeding site is reached, the bug bends the third and fourth segments of the labium backward, away from the inserted stylets until the distal section of the labium is parallel to the host surface, after which feeding can commence (Figure 12D). The bug then extracts and sucks host fluids repeatedly. Feeding may last from a few seconds to one hour at a time.

When finished feeding, the bug gradually straightens the first and second labial segments; meanwhile, the third and fourth labial segments rotate forward and contact the host surface (Figure 12E). Then the body gradually raises and pulls out the stylet fascicle (Figure 12F,G). The bug replaces the stylet fascicle into the labial groove with the help of the forelegs (Figure 12H). Finally, the rostrum rotates back to its resting position along the sternum.

The process of feeding on young shoots of the plant is similar to that observed for fruit feeding, except that the stylets are never fully retracted from the labium (Figure 11C–F).

## 4. Discussion

Substantial data are available on structure and function of mouthparts in Hemiptera. However, detail on the mechanics of feeding behavior, especially with respect to the sensory and motor feedback mechanisms, is lacking [45,46,47]. A study of the fine morphology of mouthparts allows us to interpret the function of the component parts of the feeding apparatus and improves our understanding of the actual feeding mechanism.

In this study, the feeding behavior of *E. fullo* is described. To our knowledge, this is the first time that the detailed mouthpart morphology and feeding performance in a member of Pentatomidae have been reported together. The modified mouthparts of *E. fullo* have a number of morphological similarities to those heteropteran species described previously [17,19,22,37,44,48,49,50,51,52,53,54,55], but our study revealed some new and interesting features that differ from those of other true bugs, and provide a better understanding of the feeding strategies and the sensory systems of *E. fullo*.

### 4.1. Mouthpart Morphology and Their Adaptability to Feeding

The labrum, a conspicuous anterior structure on the adult insect head, should play an important role in insect feeding. Recently the labrum was reinterpreted as fused paired appendages of an intercalary segment [56,57,58] and a few scholars have conducted detailed studies on its morphology in Heteroptera [52,53,54,55,59,60,61]. In previously published reports, the morphology of the labrum was used as a taxonomic feature of higher taxa of Heteroptera [59,60,61,62,63], but its structure varies according to feeding habits and mechanisms [61]. Spooner [59] recognized three basic types of labrum in Heteroptera: (1) a broad, flap-like labrum; (2) a long, narrow, triangular labrum; (3) a broad, flap-like sclerite with a long epipharyngeal projection. The labrum of *E. fullo* corresponds to the second group. This is similar to other true bugs, e.g., *Pyrrhocoris sibiricus* [52], *Cheilocapsus nigrescens* [53], and four species of Largidae (*Physopelta quadriguttata*, *Ph. gutta*, *Ph. cincticallis*, and *Macrocheraia grandis*) [55]. We observed regular wrinkles from base to end on the ventral surface of the *E. fullo* labrum. These wrinkles may function to add flexibility to the labrum, allowing deeper stylet penetration (Figure 12B,C). Such a long labrum (Table 1) may also be used to hold the basal part of the stylet fascicle in the labial groove during feeding (Figure 12D).

The labium of *E. fullo* has four segments as in most of other heteropteran bugs [27,52,53,54,55]. Usually, when insects are feeding, the second segment approaches the first segment, allowing the head to be lowered as the stylet fascicle penetrates the food tissue. Previous studies have reported that a band-like dorsal plate is present between the first and the second segment [27,52,54,55,64,65,66], while the base of the stylet fascicle is held in the groove of the labrum. However, in our study, there was no such structure (a band-like dorsal plate), and the distal part of the dorsum of the first segment is contracted inward (Figure 11 and Figure 12). This is probably because, unlike *Pyrrhocoris sibiricus* [52], which moves the labium back to its abdomen, *E. fullo* bends the first and second segments for deeper feeding. Moreover, the first and second segment are stronger than the third and fourth segments. The first and second labial segments of *E. fullo* are presumably folded to support the head, allowing the stylet fascicle to penetrate the plant (Figure 12B).

Heteropteran stylets form a fascicle composed of two lateral mandibular stylets and two maxillary stylets; the former are armed with teeth or rasps and the latter interlock and forms the salivary and food canals [17,18,25]. As feeding and probing on host plants are responsible for the direct or indirect damage to plants by phytophagous hemipteran insects, the stylets, including the shape and dentition of the tips, have been studied previously in several heteropterans [17,18,20,49,50,52,53,54,55,67,68,69,70,71,72,73,74,75]. In *E. fullo*, there are a series of squamous textures regularly distributed on the inner surface of the mandibular stylet and the left and right sides of the longitudinal groove are different. Similar structures are found in other phytophagous species [17,52,53,55]. Cobben [17] mentioned that the orientation of this parallel groove is such that the forward thrust of one mandible will cause considerable friction against the outer surface of the adjacent maxillary stylet contributing to its inward deviation. We also observed two nodules present on the dorsal margin of the external surface and a series of transverse ridges arranged on the outer surface. In different phytophagous Heteroptera, the nodules are slightly different, and the number of nodules also varies [49,52,53,54,55]. Depieri and Panizzai [49] observed 1 to 4 central teeth and 1–3 lateral teeth in *Dichelops melacanthus*, *Euschistus heros*, *Nezara viridula* and *Piezodorus guildinii*. Wang and Dai [52] found that mandibular stylets of *P. sibiricus* have three central teeth and two paired lateral teeth on the distal extremity, as well as five or six oblique parallel ridges on the subapex of the external convex region. In polyphagous species of Largidae (*Physopelta quadriguttata*, *Ph. gutta*, *Ph. cincticallis*, and *Macrocheraia grandis*), the serration pattern of the mandibles is 1–3 central teeth and 1–2 lateral teeth [55]. The teeth at the tip of the mandibular stylet may help to fix the stylets in host tissues [17,76].

Both mandibles together with the maxillary bundle function as a single plunging instrument [17]. Maxillary stylets are asymmetrical only in the internal positions of the longitudinal carinae and grooves. Their inner surfaces show traces of small, widely spaced notches arranged in longitudinal strips. As found by Cobben [17] in his study of *Graphosoma lineatum* L., we also found these grooves on the maxillary stylets of *E. fullo* to form a salivary canal (Sc) and a food canal (Fc). The maxillary stylets are longer than the mandibular stylets and the salivary canal is narrower than the food canal as in *Pyrrhocoris sibiricus* [52], *Cheilocapsus nigrescens* [53], *Stephanitis nashi* [54] and four species of Largidae (*Physopelta quadriguttata*, *Ph. gutta*, *Ph. cincticallis*, and *Macrocheraia grandis*) [55]. In *E. fullo*, the maxillary stylets are smooth externally but equipped with a longitudinal ridge that engages grooves in the mandibular stylets, causing it to curve inward during probing of plant tissue [17]. Moreover, the sharp ends of the maxillary stylet are specialized to pierce plant tissues while probing.

Brożek and Herczek [37] have studied the interlocking mechanisms of maxillae and mandibles in Heteroptera. Three locks between maxillae and mandibulae have been identified, i.e., dorsal, middle and ventral, similar to Fulgoroidea [3], in contrast with two locks in leafhoppers [6,8]. Our observation of the internal structure of *E. fullo* mouthparts based on the cross-section of the subapical segment of the rostrum reveals the same number of processes in each of the three locks. The food canal is oval and the salivary canal is smaller than that of the food canal, which is semicircular in cross-section. Both maxillary and mandibular stylets are flattened laterally; thus they are higher than wide in cross-section [37]. There are five upper processes on the right maxilla and six processes on the left maxilla, as found by Brożek and Herczek [37] in their study of other representatives of the Pentatomidae, e.g., *Acanthosoma haemorrhoidale* and *Elasmucha fieberi*.

Heteropteran insects have four feeding methods including stylet-sheath feeding, lacerate-and-flush feeding, macerate-and-flush feeding and osmotic pump feeding [17,28,31], and each is used on a different kind of host tissue. Miles [77] suggested that some pentatomomorphans can employ two types of feeding and that both phytophagous or carnivorous Pentatomorpha produce a stylet sheath. Generally, the polyphagous *E. fullo* primarily suck sap from the trunk, leaves, immature stems and fruits. Therefore, this species presumably employs the stylet-sheath feeding method when feeding from the phloem of the host plant, and employs lacerate-and-flush feeding when feeding on the fruit. In the stylet-sheath feeding method, the insect inserts the stylets into the feeding site (mainly phloem) and forms a salivary sheath around the stylets. In the lacerate-and-flush feeding type, these insects use their strong mandibular teeth to lacerate cells and the sharp ends of the maxillary stylet to pierce fruit for flush and suck feeding.

Usually, before feeding, heteropteran insects secrete some saliva on the surface of the host plant which is re-absorbed repeatedly to test the suitability of the feeding site [78]. In our observations, the labium lip of *E. fullo* has abundant sensilla trichodea (St3) and few sensilla basiconica (Sb5). We suspect that these large numbers of sensilla trichodea (St3) may be used to smear the saliva and the sensilla basiconica (Sb5) act as chemical sensors to taste the liquid.

### 4.2. Labial Sensillar System

Many previous authors have described rostral sensilla of Hemiptera and their possible function as chemoreceptors and mechanoreceptors [4,22,48,52,53,54,55,79,80]. Detailed morphological descriptions of Pentatomidae labial sensilla have never been previously reported. In this study, eleven types of sensilla were observed on the mouthparts of *E. fullo.*

The sensilla that cover the labial surface (except the labial tip) in *E. fullo* are evidently similar to those of most pentatomomorphan species, as well to other heteropteran species, as reported by several authors [22,44,52,53,54,55]. According to the inferred functions of the sensilla, we divided the sensilla on the labial surface into three categories: Thermo-hygroreceptive, proprioceptive and mechanosensory [43,81]. Mechanosensory sensilla include sensilla trichodea (St1, St2) and sensilla basiconica (Sb1), which have no pores or are uniporous and are embedded in flexible sockets. The proprioceptive sensilla include sensilla basiconica (Sb2), located on the junction between the first and second labial segment, and the third and fourth segment, and nonporous cupola (Sca1, Sca2) located on the surface of the cuticle or enclosed in a pit. The thermo-hygroreceptive sensilla include five types (Sb3, Sb4, Sco). Generally, all of the sensilla with this function are nonporous pegs (Sb3, Sb4, Sco).

The labial tip, which contacts the host surface during host selection and feeding, usually has poreless mechanosensory hairs and uniporous or multiporous pegs [34]. According to Rani [26], the carnivorous stinkbug *Eocanthecona furcellata* (Wolff) possesses numerous sensilla of different types at the tip of the labium, e.g., trichoid sensilla, long hairs with profusely branched shafts, an oval-shaped peg surrounded by sensory hairs with branched shafts and a short, stout peg encircled by a group of long hair-like sensilla. Six types of labial sensilla on the labium of phytophagous and predatory pentatomid species were described by Shama et al. [44]. Both studies found long cuticular projections and no sensory function on the labial tip. Nevertheless, in this study we observed in *E. fullo* many very long sensilla trichodea (St3) covering the labial tip, as well as a few sensilla basiconica (Sb5) on the central tip of the labium. Sensilla trichodea probably represent mechanosensilla as their morphology suggests, whereas sensilla basiconica are gustatory (chemosensitive sensilla). *E. fullo* is a polyphagous species sucking the sap from leaves, immature stems and fruits similar to other pentatomomorphan species. Feeding by this species causes yellowish brown spots to appear on the surface of the plant. Extensive injury results the leaf falling off. Damage to fruts includes which causes loss of edible value and yield loss [40]. So far, this is the only pentatomid species observed to have such long and numerous sensilla of the labial tip. Other studied polyphagous heteropteran species have fewer such sensilla (10 to 12 sensilla) and are more uniform in structure [22,44,52,53,54,55,82,83] in contrast to *E. fullo* in which the sensilla are much more numerous.

## 5. Conclusions

To sum up, the feeding structures in the few species of Pentatomidae studied so far seem similar to each other, presumably due to strong structural and functional constraints on their evolution. However, the mouthparts of *E. fullo* differ from those of previously studied stink bugs in the cross-sectional shape of the stylets, arrangement of labial sensilla and number of teeth of the mandibular stylets. This dissimilarity from other species of Pentatomidae and species of other hemipteran families so far described makes *Erthesina fullo* unique, particularly in its excessively long and numerous sensilla trichodea covering the end of labium. The structure and function of the mouthparts of this species are adapted for phytophagous feeding habits.

The adult feeding process involves several steps, including the exploring and puncturing of the host epidermis, a probing phase, an engorgement phase, and removal of the mouthparts from the host tissue. Studies of feeding behavior and mouthpart morphology of additional pentatomid species are needed to determine how much variation occurs in this diverse and economically important family.

## Figures and Tables

**Table 1:** Measurements of labrum and labium (mean ± SE) obtained from scanning electron microscopy. N = sample size. Lm, labrum; Lb, labium; Lb1, first segment of labium; Lb2, second segment of labium; Lb3, third segment of labium; Lb4, fourth segment of labium.

Sex	Position	Length (μm)	Width (μm)	N
Male	Lm	4687.3 ± 310.1		6
	Lb	11503.3 ± 123.9		6
	Lb1	2013.0 ± 43.4	586.7 ± 7.9	6
	Lb2	3946.8 ± 70.7	282.2 ± 5.3	6
	Lb3	3491.6 ± 118.0	384.4 ± 5.7	6
	Lb4	2366.7 ± 59.1	290.2 ± 7.6	6
Female	Lb	12952.5 ± 75.5		5
	Lb1	2359.3 ± 22.6	629.0 ± 15.5	5
	Lb2	3771.6 ± 124.4	286.3 ± 5.3	5
	Lb3	4055.9 ± 55.0	410.0 ± 6.0	5
	Lb4	2579.4 ± 19.5	315.0 ± 3.5	5

**Table 2:** Distribution, morphometric data (mean ± SE), terminology and definition of sensilla used in the present paper. Data are mean ± SE values obtained from scanning electron microscopy. N = sample number; Lm, labrum; Lb, 1, 2, 3, 4, the first, second, third, fourth segment of labium; St 1–3, sensilla trichodea 1–3; Sb 1–5, sensilla basiconica 1–5; Sco, sensilla coeloconica; Sca 1–2, sensilla campaniformia 1–2; SF, sensory field on the labial tip; Wp, wall pore; Tp, tip pore.

Type	Location on Mouthparts	Length (μm)	Basal Diameter (μm)	N	Shape	Socket	Surface	Pore	Category	Function
St1	Lm, Lb1–4	12.0 ± 1.7	1.6 ± 0.2	10	Hair	Flexible	Smooth	No	Mechanoreceptive sensilla	Tactile
St2	Lb4	79.8 ± 1.9	4.92 ± 0.2	7	Hair	Flexible	Smooth	No	Mechanoreceptive sensilla	Tactile
St3	Lb4	83.1 ± 6.3	4.2 ± 0.6	16	Hair	Inflexible	Smooth	No	Mechanoreceptive sensilla	Tactile
Sb1	Lm, Lb1–4	89.0 ± 15.4	7.4 ± 1.2	20	Hair	Flexible	Grooved	No	Mechanoreceptive sensilla	Tactile
Sb2	Lb2, Lb 4	86.3 ± 7.1	6.5 ± 0.7	6	Peg	Flexible	Smooth	Wp (Uniporous)	Proprioceptive sensilla	Perceive the degree of flexion of the joint
Sb3	Lb2	55.8 ± 3.3	6.8 ± 0.4	16	Peg in pit	Inflexible	Smooth	No	Thermo-hygroreceptive sensilla	Temperature/humidity
Sb4	Lb4	12.8 ± 0.6	1.9 ± 0.1	6	Peg in pit	Inflexible	Smooth	No	Thermo-hygroreceptive sensilla	Temperature/humidity
Sb5	SF	Longer than Sb1	Wider than Sb1		Peg	Flexible	Smooth	Tp	Chemoreceptive sensilla	Gustatory
Sco	Lm, Lb1–4		2.4 ± 0.4	10	Pegs in cavity	Inflexible	Smooth	No	Thermo-hygroreceptive sensilla	Temperature/humidity
Sca1	Lb1, 2, 4		6.7 ± 0.4	4	Oval plate	Inflexible	Smooth	No	Proprioceptive sensilla	Perceive the degree of flexion of the joint
Sca2	Lb4		6.2 ± 1.2	4	Oval plate	Inflexible	Smooth	No	Proprioceptive sensilla	Perceive the degree of flexion of the joint
